# Temperature Dependence of Water Contact Angle on Teflon AF1600

**DOI:** 10.1021/acs.langmuir.1c03202

**Published:** 2022-01-20

**Authors:** Yijie Xiang, Paul Fulmek, Daniel Platz, Ulrich Schmid

**Affiliations:** Institute of Sensor and Actuator Systems, Vienna University of Technology, Vienna 1040, Austria

## Abstract

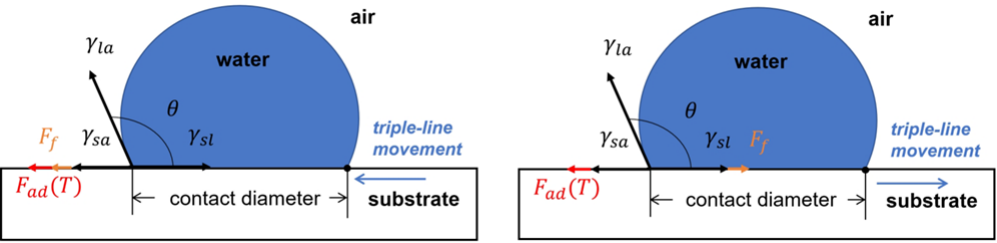

In this work, we investigate the change of contact angle (CA) of a water droplet during evaporation on a Teflon AF1600 surface in the temperature range between 20 and 80 °C under standard laboratory conditions. An almost constant initial CA and a significant increase of the stabilized CA have been observed. The results reveal a temperature-dependent CA change, mainly due to water adsorption on the solid surface. Soaking experiments indicate that besides adsorption, a temperature-independent friction-like force contributes to the pinning of triple-line and therefore to the CA change. We propose an adsorption coverage parameter and a friction-like force to describe the CA change. Furthermore, we describe a reproducible process to produce smooth and homogeneous Teflon AF1600 thin films, minimizing the influence of roughness and local heterogeneity on the CA.

## Introduction

Wettability of a droplet on a solid surface is an essential property of surface science. The primary method to describe the wettability is the contact angle (CA) measurement. The CA is defined by Young’s equation,^1^ as the angle θ_Y_ at the triple-line governed by equilibrium of surface tensions
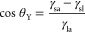
1where γ is the interface tensions, and the subscripts *s*, *l*, *and a* represent solid, liquid, surrounding air, respectively. Changing and controlling the surface wettability and CA by microstructure-patterning for achieving superhydrophobic surface (Lotus Effect^2^), applying voltage for manipulation of conductive liquids on a surface (Electrowetting^3−5^), and so forth has been a research hotspot over the last decades. However, variations from θ_Y_ are observed. These variations lead to an inaccurate control of the CA and surface wettability.

Standard explanations for the variation of CA from θ_Y_ are surface imperfections, such as surface roughness,^6^ chemical heterogeneity,^7^ and adsorption.^8^ The Wenzel-state has been introduced to describe the CA on a rough surface.^9^ It indicates that the surface roughness increases the hydrophobicity if the surface is hydrophobic, that is, CA > 90°. In contrast, roughness enhances the wettability of hydrophilic surfaces, that is, CA < 90°. Another expansion of Young’s equation, known as Cassie’s equation, takes the local chemical heterogeneity of the surface into account.^10^ Moreover, molecule adsorption on the solid surface also significantly affects the solid surface’s energy, and consequently the CA. Water molecules can physically and chemically adsorb on all solid surfaces with high surface energy (hydrophilic surfaces).^11^ Water molecules can also adsorb onto hydrophobic materials, like Teflon.^12−16^ Lam et al.^8^ experimentally demonstrate the influence of chain length and molecule size of the liquid on the adsorption process and therefore the CA change: the smaller the molecule size of the liquid is, the higher is the possibility of being adsorbed on the surface, causing a more significant CA change.

The droplet evolution during the evaporation process on solid surfaces has been studied for years, a significant CA change from its initial value has been observed. Usually two regimes of droplet evolution are identified: a constant contact radius (CCR), and a constant contact angle regime (CCA).^8,17−20^ In the CCR regime, the contact area stays constant, meanwhile, the CA decreases during droplet volume loss by evaporation. When the CA reaches a specific value, the CCR regime transforms to the CCA regime, in which a constant CA is observed. Therefore, two distinct values of the CA are identified: the initial CA, and the receding or stabilized CA.^21,22^ For inhomogeneous solid surfaces, an additional regime of stick–slip has been observed.^8,23^

Teflon AF1600 is a amorphous fluoropolymer based on a copolymer of 4,5-difluor-2,2-bis(trifluoromethyl)-1,3-dioxol (PDD, 65 mol %) and tetrafluoroethylene (TFE). It has extraordinary properties, such as high-temperature stability,^24^ outstanding chemical resistance, and low surface energy, widely used in application fields requiring a high water contact angle (WCA), for example, EW-based variable focus liquid lens^4,5^ and so forth. Compared to traditional fluoropolymers, such as polytetrafluoroethylene (PTFE) and perfluoroalkoxy alkane (PFA), which are mostly semicrystalline or highly crystalline and only soluble in high-boiling solvents at a temperature near their melting point,^25^ Teflon AF1600 is amorphous and soluble in some fluorinert solvents at room temperature. Because of its solubility, Teflon AF1600 can be used to make thin and homogeneous films by various methods, including spin coating, dip coating, and spray coating.^25,26^

In this work, we investigated the WCA change on a Teflon AF1600 surface by water evaporation at various temperatures and observed the evolution of droplets and WCA with time. We compared the WCA change at various temperatures and conducted soaking experiments to observe the influence of water adsorption on the WCA. From these experiments, we concluded that the WCA change by evaporation is mainly due to a temperature-independent friction-like force and the temperature-dependent water adsorption on the solid surface. Finally, we proposed a compact model to describe the WCA change.

## Experimental Details

### Fabrication of Thin Film

A reproducible fabrication process is presented to achieve smooth, pinholes-free Teflon AF1600 thin films to minimize the influence of roughness and local heterogeneity on the CA.

**Figure 1:**
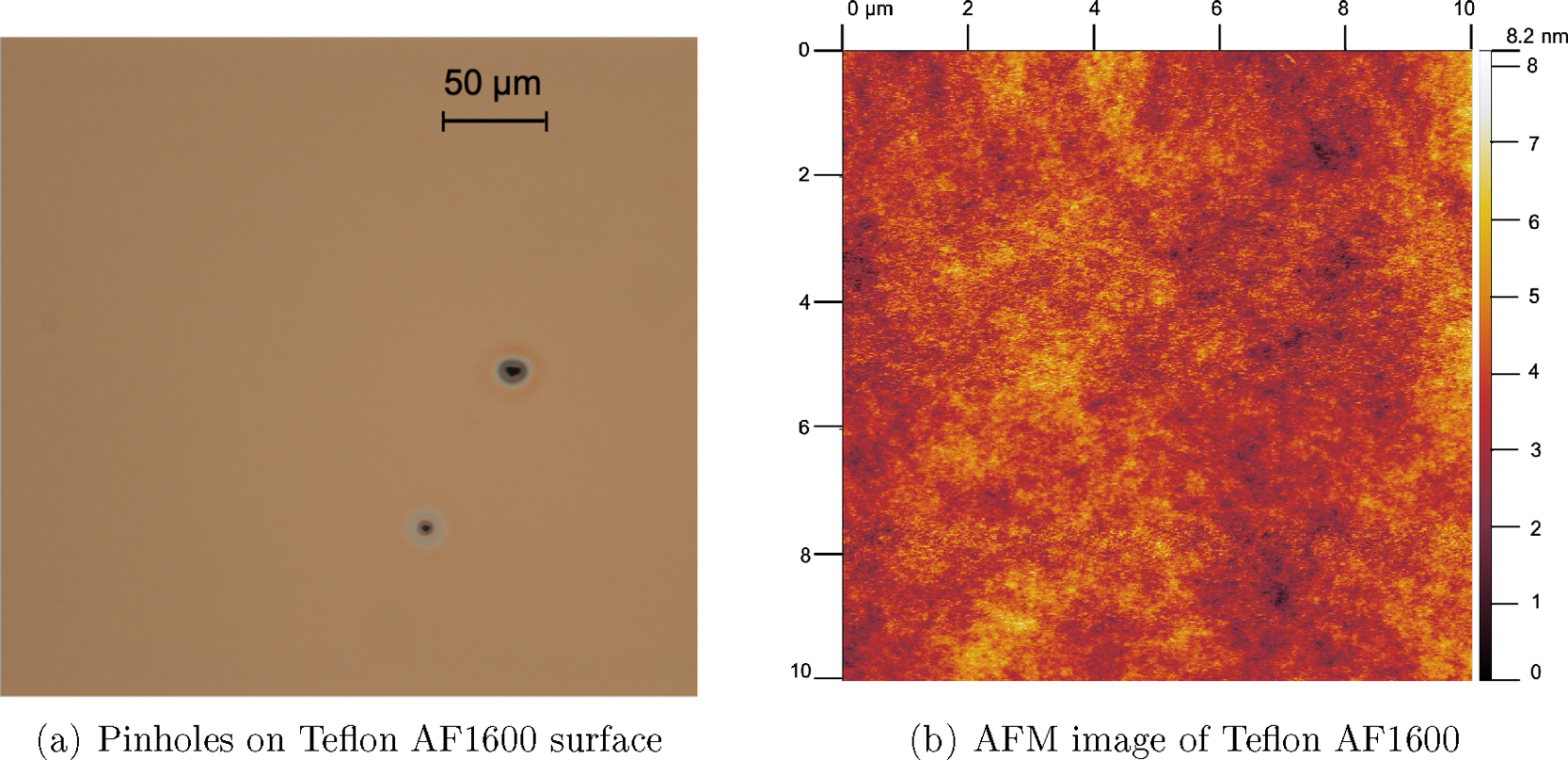
(a) Optical micrograph of a Teflon AF1600 surface: pinholes are observed after the third baking step with 330 °C. (b) A typical AFM image of Teflon AF1600 surface fabricated by two baking temperatures. The mean root square roughness is 0.82 nm.

A 2% solution by weight of Teflon AF1600 (DuPont Co. U.S.A.) in fluorinert FC-40 solvent (3M Company) is prepared and stirred at 50 °C for 4 days. The solution is then filtered using a PTFE membrane (pore size 5 μm, purchased from Sigma-Aldrich) to remove undissolved or aggregated particles. After filtration, the solution is placed in vacuum for a few minutes to remove air bubbles, which are trapped in the solution or created by the filtration process. In order to increase the adhesion of Teflon AF1600 on the solid substrate, titanium (Ti) prime is first spun at 4000 rpm for 1 min onto a silicon wafer (spin coater, Süß Technic) and dried at 120 °C for 2 min. The Teflon AF1600 solution is then spun at 4000 rpm for 1 min. Next, the samples are soft-baked on a hot plate at 175 °C for 10 min to remove the solvent, followed by two additional hard-baking processes at 165 °C (glass transition temperature of Tefon AF1600) and 330 °C (glass transition temperature of PDD) for 5 and 15 min, respectively. This baking procedure is the standard process proposed by DuPont. However, pinholes have been observed on the surface after the third baking step, seen in Figure 1a, and an additional stick–slip phenomenon appeared in our preinvestigation of the WCA during evaporation.

To investigate the influence of the third baking step, we used different methods to determine the surface quality before and after the last baking with temperature of 330 °C: Fourier-transform infrared spectroscopy^24^ (FTIR, Tensor Bruker 27, reflective spectral range of 3500–400 cm^–1^) for analyzing the surface functional groups (the FTIR results are shown in Supporting Information), atomic force microscopy (AFM, Bruker Dimension Edge, with Bruker NCHV-A cantilever in tapping mode) for observation of surface morphology, and WCA measurement (Krüss DSA30S, sessile droplet method) to estimate the surface tension. These properties of the Teflon AF1600 thin film showed no changes after the last baking step.

Therefore, we have chosen two baking steps at 175 and 165 °C for 10 and 5 min, respectively, to achieve pinhole-free Teflon AF1600 surfaces. The average thickness 210 ± 15 nm of the Teflon AF1600 film was determined by measuring the step height with a profilometer (Dektak). The surface roughness was determined by AFM and resulted in a mean root square roughness *R*_q_ = 0.82 nm on a scan area of 10 × 10 μm^2^. A typical AFM image of Teflon AF1600 is shown in Figure 1b.

### Experimental Setup

**Figure 2:**
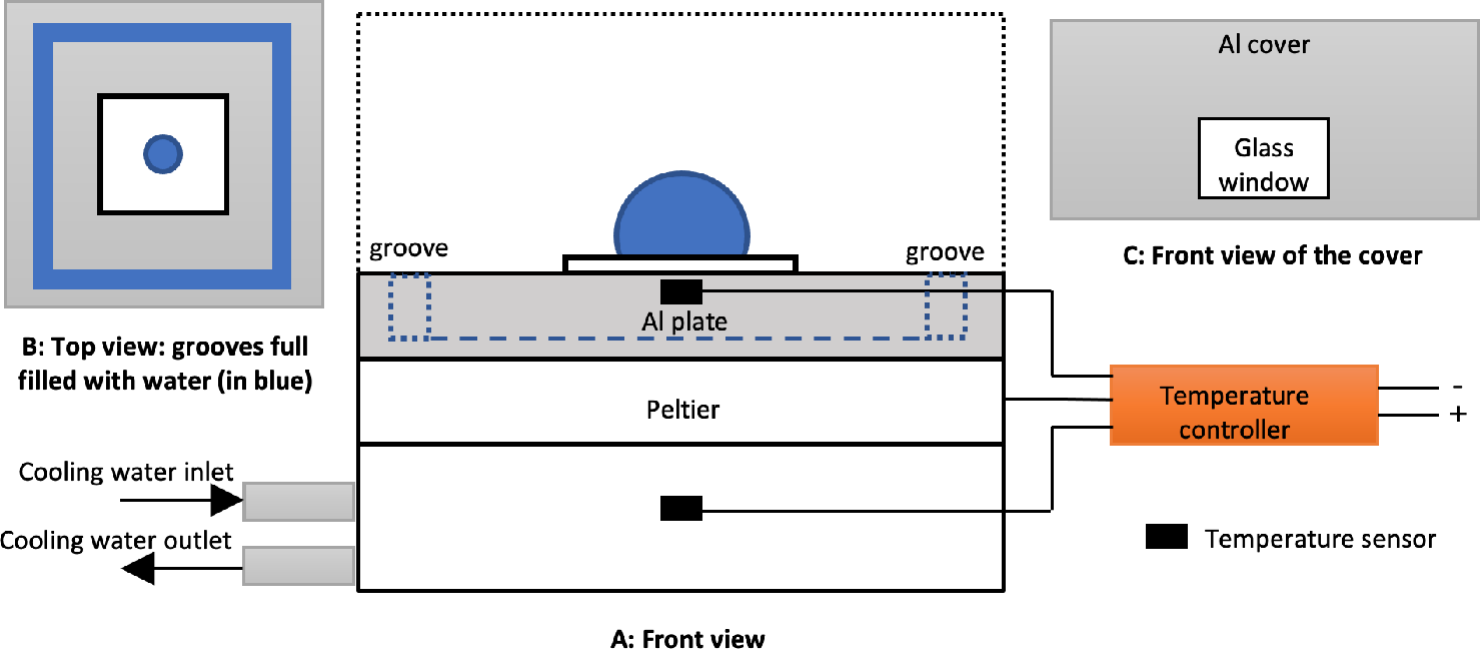
Illustration of the experimental setup. (A) Front view, (B) top view, and (C) front view of the Al cover with glass windows and a small opening with 2 mm diameter at the top for inserting dosing needle. A Peltier element under the Al plate, two temperature sensors, and a temperature controller guarantee a homogeneous temperature contribution. For water droplet evaporation process, the Al cover (black dash line in A) is removed to achieve atmospheric humidity and allowing droplet evaporation. For soaking experiments to investigate the effect of water adsorption on WCA, the groove integrated in the Al base plate is filled with water to create an atmosphere of saturated humidity (blue dash line in A and details seen in B), and the Al cover is placed to maintain the saturated humidity inside the chamber.

The contact angle measurements are carried out under standard chemical laboratory conditions, that is, constant temperature of 20 ± 1 °C, atmospheric pressure and relative humidity of 35 ± 1%. The experimental setup consists of a drop shape analyzer (DSA, Krüss DSA30S) and a custom-built temperature controlled test chamber (see Figure 2). Images with a resolution of 200 Pixel/mm taken from DSA are analyzed for the droplet contour. The pendant drop method is used to measure the liquid surface tension, while the sessile drop method is used to determine the CA.^27^ The measurement resolutions are 0.01° for the CA, and 0.01 mN/m for the surface tension. At the baseplate the temperature is precisely controlled (*ΔT* < ±0.01 °C) by a Peltier element with a controller (Meerstetter TEC-1091). The high thermal conductivity of the aluminum (Al) plate guarantees a homogeneous temperature distribution at the baseline of the droplet as well as at the triple-line. The Al-cover reduces the *T* gradient in the chamber. A groove inside the chamber, which can be filled with water, allows to control the humidity inside the chamber and to reduce the evaporation speed. At a chamber temperature of 20 ± 1 °C the evaporation rate has been reduced from 0.16 to 0.004 μL/min.

As evaporation experiments can last for 1000 s and more, we preinvestigated if contaminations of the water droplet from the surrounding air affect measurement results. The pendant drop contour^27^ was used to determine the surface tension of deionized water (DI water, 16–18 MΩ·cm). The result showed that the water surface tension decreased by less than 1 mN/m from 72.8 to 72 mN/m after 1500 s at laboratory condition. For our experiments this would result in a WCA change of less than 0.5°. On the basis of these results, we neglect the influence of contamination.

Droplet evolution and spreading on solid surfaces is governed by capillary, triple-line, and viscous force. The capillary number Ca is used to describe the relative effects of viscous forces and surface tension forces acting across the boundary: Ca = μ·*v*/γ^28^ with the dynamic viscosity of the liquid μ, the spreading velocity *v* of the contact line, and the liquid surface tension γ. The maximum contact line velocity observed in our experiments results in a maximum capillary number 7 × 10^–8^, which is substantially lower than the critical value of 10^–5^,^29^ therefore any impact of viscous force is neglected in this work.

## Results and Discussion

### Contact Angle during Droplet Evaporation

**Figure 3:**
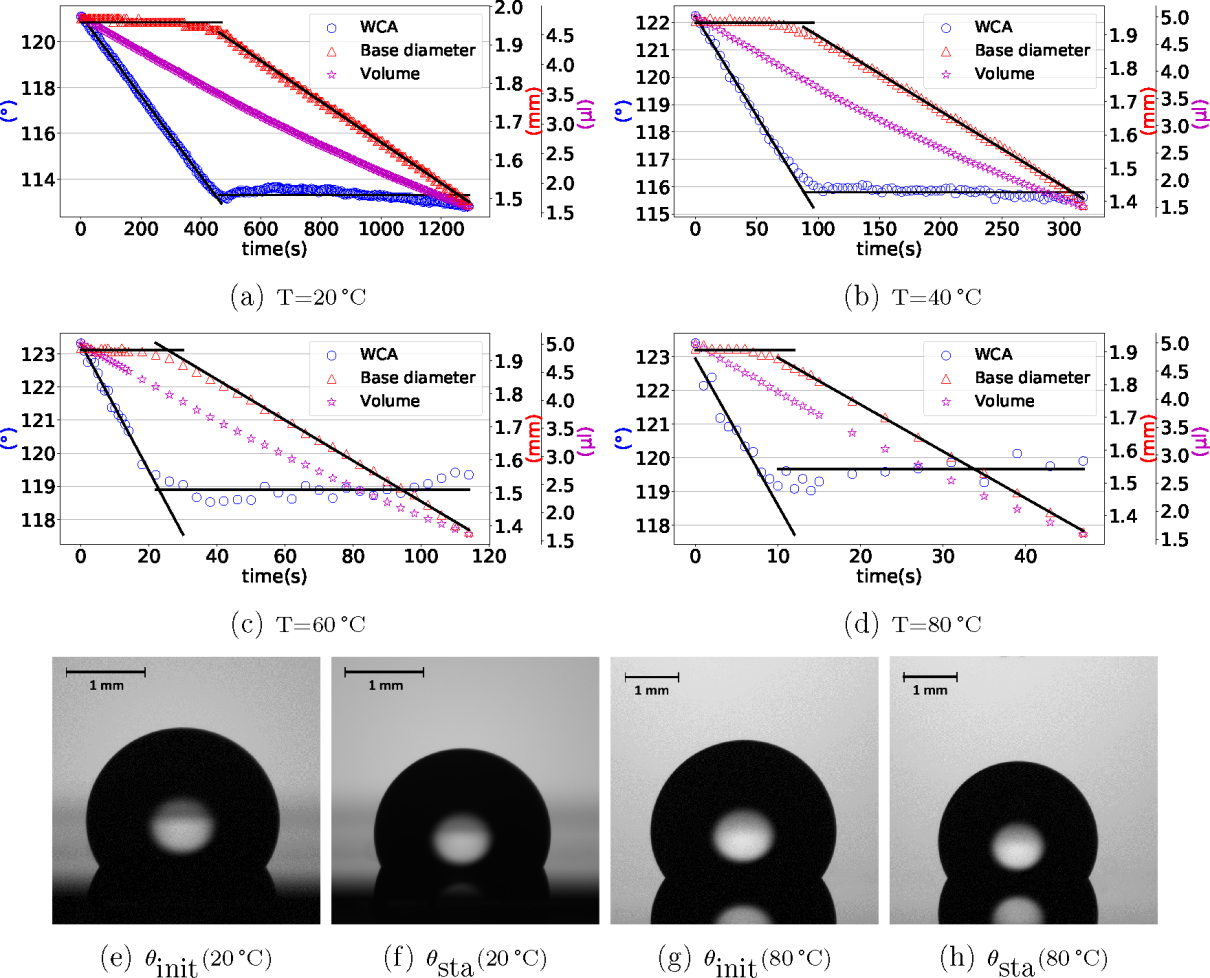
WCA, contact base diameter, and droplet volume evolution due to droplet evaporation over time at (a) *T* = 20 °C, (b) *T* = 40 °C, (c) *T* = 60 °C, (d) *T* = 80 °C. The solid lines represent the fitted curves in the CCR and CCA regime. In the CCR regime, the contact base area stays constant and the WCA decreases. In the CCA regime, however, the contact base area decreases and the WCA stays constant. Representative droplet images of (e) initial WCA θ_init_ at *T* = 20 °C, (f) stabilized WCA θ_sta_ at *T* = 20 °C, (g) initial WCA θ_init_ at *T* = 80 °C, (H) stabilized WCA θ_sta_ at *T* = 80 °C.

First, the sample is cleaned with acetone and isopropanol, and then blown dry with nitrogen to ensure a clean and dry Teflon AF1600 surface. Next, the temperature of the base plate is adjusted. A droplet with a volume of 5 μL is generated at the needle tip, and then slowly deposited onto the sample surface. The WCA measurement starts after ∼1 s, when the droplet has reached its thermal equlilibrium at the sample surface. Within the first 15 s, images of the droplet are taken every second, and then the droplet shape evolution is monitored every 4 s. The measurements stop when the drop volume is less than 1.5 μL. Figure 3 shows typical results for the droplet evaporation process over time: the evolution of the WCA, the droplet volume, and the contact area diameter at four temperatures. It shows clearly a CCR regime followed by a CCA regime. In the CCR regime, the triple-line stays pinned at a fixed position (constant contact base diameter) while volume and WCA continuously decrease. As the triple-line gets depinned and starts moving, the CCA regime is established and the WCA stays constant while the droplet evaporates. We extract two characteristic WCAs: the initial WCA (θ_init_) and the stabilized WCA (θ_sta_). Both values are determined from the experimental data by a linear least-square fit.

**Figure 4:**
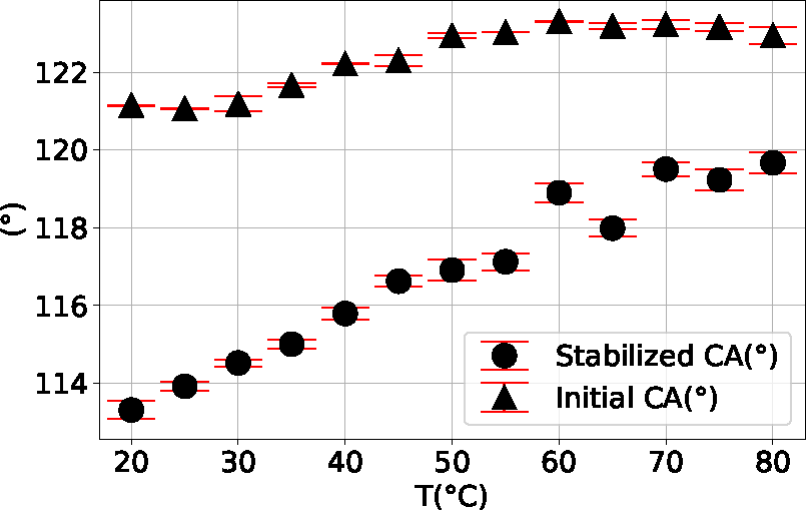
Measurement results for θ_init_ and θ_sta_ of DI-water on Teflon AF1600 over temperature.

Figure 4 shows the initial and stabilized WCAs for the temperature range from 20 to 80 °C. The initial WCA θ_init_ is almost constant, changes slightly from 121.1° to 122.9°, and the stabilized WCA θ_sta_ shows a significant increase from 113.3° to 119.7°. This trend is also observed by Weisensee et al.,^13^ who have controlled the pressure to the corresponding temperatures. The uncertainty of the measurement of the initial WCA, especially for temperatures >50 °C with evaporation rates >0.02 μL/s, can be determined from the maximum measurement delay of 1 s: the measured θ_init_ could be maximal 0.3° smaller than the actual θ_init_. Moreover, the θ_sta_ at higher temperatures (*T* > 50 °C) are more scattered with a standard deviation of ∼0.25° compared to ∼0.12° at lower temperatures (*T* < 50 °C).

**Figure 5:**
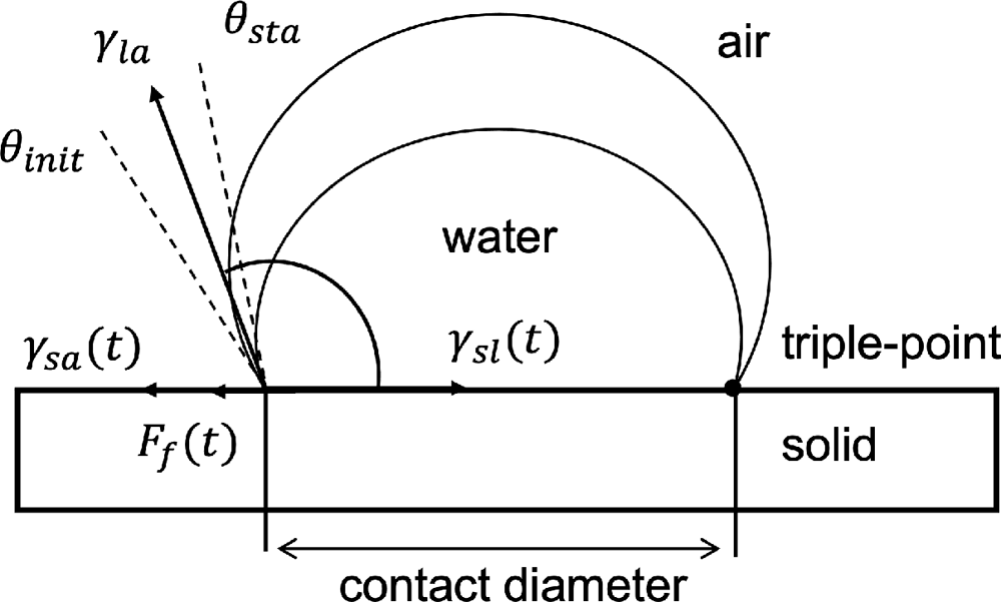
Force equilibrium at the triple-line. Interface tensions γ_la_, γ_sl_, γ_sa_ and a friction-like force *F*_f_ contribute to the force equilibrium.

**Figure 6:**
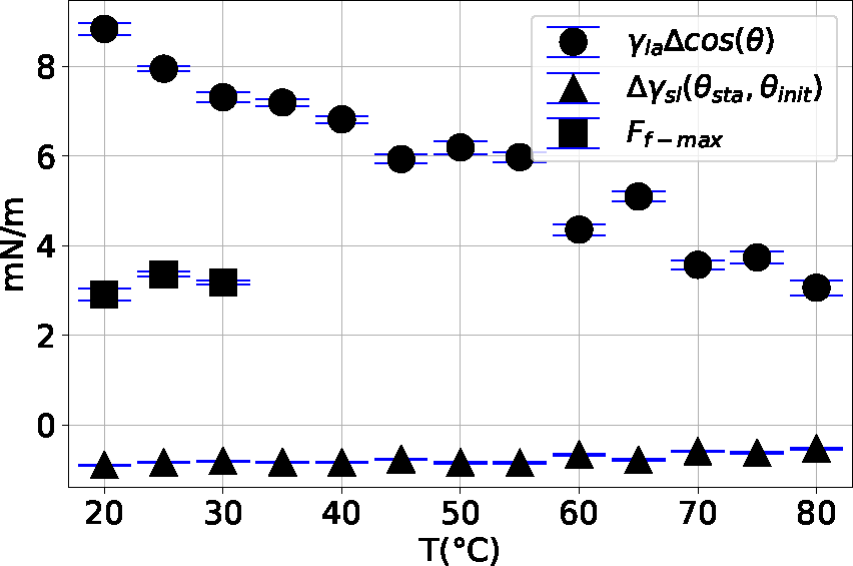
Results of effective solid surface tension change γ_la_·Δcos θ from evaporation experiments, the solid–liquid interface tension difference *Δγ*_sl_(θ_sta_, θ_init_) calculated from Gibbs adsorption equation, the friction-like force *F*_f-max_ evaluated from soaking and evaporation experiments. All results are plotted against temperatures.

According to the Young’s equation (eq 1) the change of the quasi-static WCA is governed by the interface tensions equilibrium: γ_la_·cos θ = γ_sa_ – γ_sl_. As γ_la_ stays constant during the evaporation process at a constant temperature and pressure, the variation of the WCA θ_init_ → θ_sta_ is due to the changes of γ_sa_ and γ_sl_, consequently
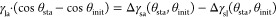
2with *Δγ*_s*_(θ_sta_, θ_init_) = γ_s*_^sta^ – γ_s*_^init^, the subscript s* represents sl and sa. The values of the temperature dependence of γ_la_ is taken from literature^30^ and validated by some own experiments. The change of γ_sa_ is mainly due to the adsorption of water onto surface, which can be reduced by temperature. Figure 4 shows that at *T* = 80 °C the effective solid surface tension change calculated from experimental data by γ_la_·(cos θ_sta_ – cos θ_init_) is ∼3 mN/m. This change can not be explained by the adsorption of water on a hydrophobic surface at such high temperature. More details are discussed in the following soaking experiment part. Besides γ_la_ and γ_sa_, the solid–liquid interface tension γ_sl_ also contributes to the force equilibrium at the triple-line. The difference of the solid–liquid tension *Δγ*_sl_(θ_sta_, θ_init_) is described by Gibbs adsorption equation^31^
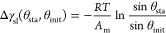
3The results for *Δγ*_sl_(θ_sta_, θ_init_) are depicted in Figure 6 with the universal gas constant *R*, the absolute temperature *T*, and the molar surface area of Teflon AF1600 *A*_m_ (details seen in Supporting Information). The *Δγ*_sl_(θ_sta_, θ_init_) calculated by Gibbs adsorption equation cannot sufficiently explain the change calculated by Young’s equation from experimental data γ_la_·(cos θ_sta_ – cos θ_init_). Consequently, we assume an extra force *F*_f_ with static friction-like behavior (see Figure 5) to extend the Young’s equation (eq 1) ensuring the static force equilibrium
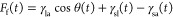
4The WCAs are observed over time during the evaporation process.

When the droplet is deposited on the solid surface, it adjusts to an equilibrium shape by the initial interface tensions with an initial contact radius and an initial WCA θ_init_: *F*_f_^init^ = γ_la_ ·cos θ_init_ + γ_sl_^init^ – γ_sa_^init^. The superscript init refers to the initial solid surface condition (dry) with the corresponding WCA θ_init_. By considering the droplet diameter in this work (∼2 mm, smaller than the capillary length of water 2.7 mm) and the deposition method, we assume *F*_f_^init^ = 0. While the WCA is decreasing, the friction-like force increases up to its maximum value *F*_f-max_ when the triple-line is depinned and starts moving in CCA mode with the corresponding θ_sta_. The force equilibrium is then *F*_f-max_ = γ_la_·cos θ_sta_ + γ_sl_^sta^ – γ_sa_^sta^, where the superscript sta refers to the solid surface condition in the absorption–desorption equilibrium state around the triple-line with the corresponding WCA θ_sta_. Combining the force equilibrium equations, we have
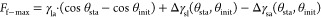
5where *Δγ*_sl_(θ_sta_, θ_init_) can be calculated by eq 3 with θ_init_, θ_sta_ and the corresponding *T*.

### Contact Angle by Soaking Experiments

From the evaporation experiments, we know that the triple-line movement for the receding WCA leads to the direction of the water covered surface, previously below the droplet. Accordingly, the solid–air interface tension γ_sa_ should have experienced the influence of liquid water adsorption. The change of γ_sa_ can be calculated based on Gibbs adsorption equation as^16^*Δγ*_sa_ = *RT* ∫_0_^*p*_0_^ Γd ln *p*_v_, where Γ is the concentration of adsorbed vapor on a solid as a function of the vapor pressure *p*_v_ up to its equilibrium vapor pressure *p*_0_ of the liquid. The concentration of adsorbed vapor Γ is hard to be estimated and also hard to be directed measured in situ for hydrophobic materials. Consequently, we tried to directly analyze this effect by another WCA experiment on a sample surface that has been completely soaked in DI water.

First, we immersed the well-cleaned samples into DI water for 10 min up to 6 h at *T* = 20, 25, 30 °C allowing the water to adsorb onto Teflon AF1600. The WCAs on the soaked samples were then measured in the chamber at the same temperature (see Figure 2), while the groove was filled with DI water to ensure a saturated humidity and therefore prevent the desorption and evaporation. The immediately following WCA (θ_soak_) experiments showed a significant reduction of 4–5° compared to the initial WCA (see Table. 1), while the immersion time had no significant effect on the WCA reduction. In a second step, we tried to reverse the adsorption process by heating the sample surface to 50 °C for 10 s and blowing with nitrogen separately. The followed WCA experiments on those surfaces again showed the expected large initial WCA.

**Table 1:** Soaking Experiment Results^a^

*T*	θ_init_	θ_soak_	θ_sta_	F_f-max_
20	121.1 ± 0.01	116.1 ± 0.05	113.3 ± 0.23	2.91 ± 0.13
25	121.0 ± 0.01	117.2 ± 0.03	113.9 ± 0.10	3.36 ± 0.06
30	121.2 ± 0.20	117.7 ± 0.08	114.5 ± 0.09	3.18 ± 0.05

a The effective solid energy change after soaking γ_la_·(cos θ_soak_ – cos θ_init_) 5.58 mN/m for 20 °C is comparable with the immersion energy 6 mN/m of Teflon measured by Chessick et al.^14^.

On the basis of the results, we conclude that the physical and reversible adsorption of water on the Teflon AF1600 surface is a dominating reason for the WCA reduction at low temperature. With the experimental results for the θ_init_ on the dry surface and the θ_soak_ on the soaked surface, we get the WCA change by using eq 4
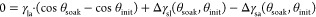
6where *Δγ*_sl_(θ_soak_, θ_init_) can be calculated by eq 3. *Δγ*_sa_(θ_soak_, θ_init_) = γ_sa_^soak^ – γ_sa_^init^, equals to *Δγ*_sa_(θ_sta_, θ_init_) = γ_sa_^sta^ – γ_sa_^init^ with the consideration that the γ_sa_^soak^ and γ_sa_^sta^ are both referring to the solid surface that is covered with water before and in adsorption equilibrium. With the experimental results of the stabilized WCA θ_sta_, we evaluate eq 5 and eq 6 to find the force *F*_f-max_
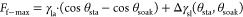
7The results are listed in Table 1. The value of *F*_f-max_ is almost constant 3.15 ± 0.19 mN/m, independent of temperature. The effect of adsorption on the WCA decreases with temperature and is very limited at temperature higher than 50 °C. The effective solid surface tension change at 80 °C, γ_la_·(cos θ_sta_ – cos θ_init_), is mainly due to the friction-like force, which is consistent with the evaporation experimental result, ∼3 mN/m.

## CA Model

From the experimental results, we identified two contributions to the force equilibrium at the triple-line: a temperature-independent friction-like force and a temperature dependent adsorption on solid surface. The adsorption is hard to measure quantitatively in situ for hydrophobic materials. We therefore propose a model to describe the WCA considering the adsorption coverage *f* on the solid surface. The adsorption coverage is defined as the area fraction of the occupied adsorption area to the total adsorption area.^32^ The energy contribution of a fully occupied adsorption area is *e* with the same unity of γ. The surface energy for partial adsorption on the solid can be written as
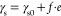
8where γ_s0_ is the solid surface energy in vacuum, originating from the surface relaxation and reconstruction. It represents the water adsorption-free surface. The interface energy between the liquid droplet and the solid surface is assumed as γ_sl_ = γ_s0_ + *e* with 100% coverage (*f* = 1) based on the fact that the γ_sl_ change during evaporation is limited. The solid–air interface energy for the dry hydrophobic surface is assumed as γ_sa_^init^ = γ_s0_ with 0% coverage (*f* = 0). When the adsorption–desorption process reaches its equilibrium, the solid–air interface energy can be written as γ_sa_^sta^ = γ_s0_ + *f* · *e* with a equilibrium coverage *f*. Plugging those conditions into eq 4, the initial WCA and stabilized WCA are described by
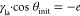
9and
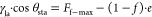
10respectively. The relation between the initial WCA and the stabilized WCA is then given by
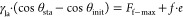
11The friction-like force *F*_f-max_ is evaluated from soaking experiments, considered as temperature independent. *e* can be calculated from eq 9. It shows a constant *e* value in the temperature range from 20 to 50 °C with *e* = 37.15 ± 0.26 mN/m. The adsorption coverage *f* over temperature can be therefore calculated with eq 11. The model shows the expected low water adsorption ability of Teflon AF1600. The reachable adsorption coverage at 25 °C is 0.12. This value is comparable to the earlier results.^13−16^ At temperatures higher than 50 °C, the coverage approaches zero.

## Conclusion

In this work, we investigated the WCA change during evaporation as a function of temperature and conducted soaking experiments to explore the influence of adsorption. We evaluated two main contributions for the WCA variation: a friction-like force, which has a constant value of 3.15 ± 0.19 mN/m and is temperature independent, and a contribution of water adsorption on the solid near the triple-line, which decreases with increasing temperature and completely vanishes at temperatures higher than 50 °C. Besides these basic findings, this knowledge is also for application driven developments, such as the EW-based manipulation of droplets, of high interest. If, for instance, the triple-line moves to the adsorption-free area by applying voltage (EW application) or by increasing the droplet volume (establishing of the advancing CA), the CA variation is dominated by the friction-like force. If the triple-line moves to the water-absorbed or water-covered area when turning-off the applied voltage (EW application) or by decreasing the droplet volume (establishing of the receding CA), the friction-like force and the contribution of water adsorption are both responsible for the CA variation and need to be taken into account for a precise determination of the corresponding CA. Additionally, an increasing temperature can reduce or completely remove the adsorption-induced CA variation. Furthermore, we introduced an adsorption relative parameter coverage *f*, friction-like force limit *F*_f-max_ and propose an adsorption-area density dependent model to describe the WCA change. Our proposed model is valid for hydrophobic materials, where discrete adsorption sites are available and the adsorption does not form continuous films. This simple model also provides a possibility to estimate the water adsorption coverage by measuring the WCA change, since the adsorption quantity and coverage on a hydrophobic thin film is difficult to be directly measured.

In further work, the proposed model and the results of WCA at different temperatures will be carefully expanded for EW-based applications, thus paving the way for an accurate prediction of droplet manipulation under different environmental conditions.

## Supporting Information Available

The comparison of FTIR results of Teflon AF1600 fabricated with and without the third baking step; details for derivation of eq 3 and the calculation of *A*_*m*_ of Teflon AF1600 (PDF)
