# Supplementary material for: Temperature Dependence of Water Contact Angle on Teflon AF1600
Source: Langmuir. 2022 Jan 20;38(4):1631–7. doi: 10.1021/acs.langmuir.1c03202 (PMC8812120; doi:10.1021/acs.langmuir.1c03202)
Supplement: Supplementary file 1 — la1c03202_si_001.pdf [file la1c03202_si_001.pdf]

SUPPORTING INFORMATION

Temperature dependence of water contact angle on Teflon AF1600

Yijie Xiang,* Paul Fulmek, Daniel Platz, and Ulrich Schmid*

Institute of Sensor and Actuator Systems, Vienna University of Technology, Vienna, 1040, Austria

E-mail: yijie.xiang@tuwien.ac.at; ulrich.e366.schmid@tuwien.ac.at

Contents

Comparison of FTIR results **2**

Derivation of the Eq. (3) and the calculation of A_m **3**

Comparison of FTIR results

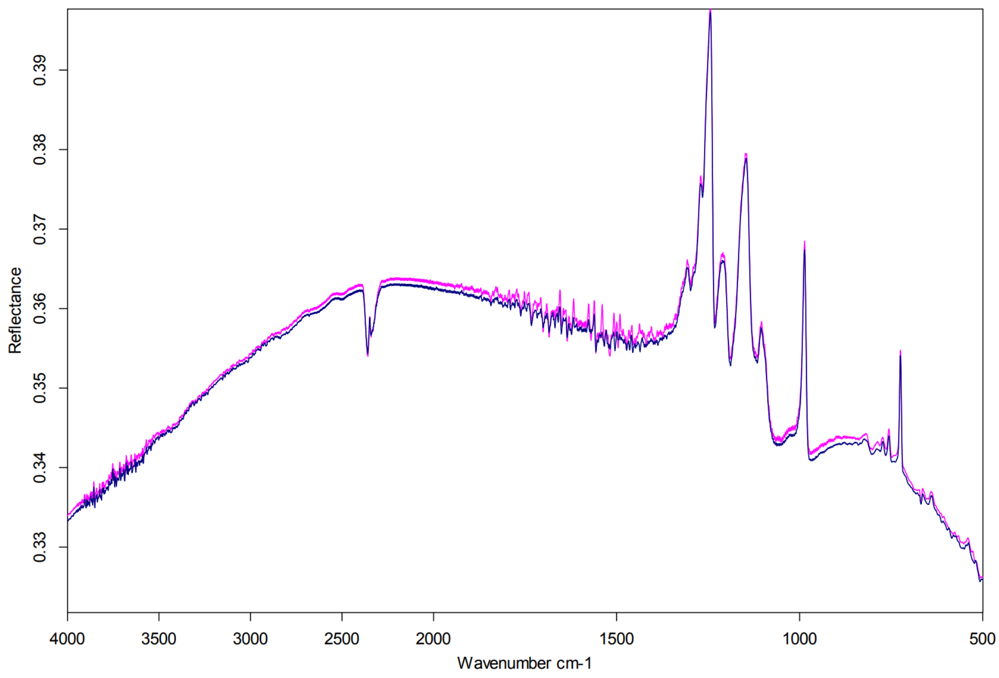

Figure S1: The FTIR spectra of Teflon AF1600 with two baking steps (in violet) and with three baking steps (in blue). The peaks at 2000 $\sim$ 1500cm^{-1} and $\sim$ 2300 cm^{-1} indicate water and carbon dioxide inside the measurement chamber, respectively. In the interesting wavenumber range between 1500 to 500 cm^{-1} for Teflon AF1600, no differences have been found between both spectra within the measurement accuracy.

Derivation of the Eq. (3) and the calculation of A_m

An expression based on the Gibbs adsorption equation has been presented by *Extrand*[1] to characterize the energy change of the solid-liquid interface between advancing and receding WCA. We use the same expression to describe the energy change during the evaporation process, from the initial WCA to the stabilized WCA: $\Delta g = -(\frac{RT}{A_m}) \ln \frac{\sin\theta_{\text{sta}}}{\sin\theta_{\text{init}}}$. The A_m is the molar surface area of solid surface and calculated by:

$$A_m = (\frac{M_0}{\rho})^{2/3} N^{1/3}. \tag{1}$$

In our case, the solid material is Teflon AF1600. The A_m of Teflon AF1600 is 192 436.08 $\mathrm{m^2/mol}$, calculated with the Avogadro constant $N = 6.022 \cdot 10^{23} \mathrm{mol^{-1}}$, the density $\rho = 1.78\,\mathrm{g/mL}$, and the monomer weight M_0, given by the components 4,5-difluor-2,2-bis-47(trifluormethyl)-1,3-dioxol (PDD, 244.04 g/mol, 65 mol %) and tetrafluorethylen (TFE, 100.02 g/mol, 35 mol %), $M_0 = 193.63\,\mathrm{g/mol}$.

A short derivation of the expression Δg achieved by *Extrand* is introduced in the following, and more details can be found in the original paper. The Gibbs adsorption equation relates the surface free energy g of the solid to its chemical potential μ_s:

$$-\mathrm{d}g = \frac{1}{A_m} \mathrm{d}\mu_s. \tag{2}$$

At the solid-liquid interface, the chemical potential change of the liquid-molecules adsorbed onto the solid μ_s is equal to that of the liquid droplet μ_l:

$$\mathrm{d}\mu_s = \mathrm{d}\mu_l = RT\mathrm{d}(\ln p_l) \tag{3}$$

where R is the ideal gas constant, T is the absolute temperature and p_l is the pressure across the liquid interface. The Δg between the solid states with initial WCA and the stabilized

WCA can be therefore written by combining Eq. 2 and Eq. 3 as:

$$\Delta g = g^{sta} - g^{init} = -\frac{RT}{A_m} \ln \frac{p_l^{sta}}{p_l^{init}}. \tag{4}$$

p_l^{sta} and p_l^{init} can be calculated by Young-Laplace pressure,[2]

$$p_l = \gamma_{\text{la}} \cdot \frac{2}{r}, \tag{5}$$

with the radius r of the liquid droplet. Assuming a spherical cap shape of the small droplet in our case ($r = 1\,\text{mm}$), r can be written as

$$r = \frac{x}{\sin\theta}, \tag{6}$$

where x is the contact area radius. In the CCR regime, the triple-line is pinned, so that the contact area radius x is constant, while the WCA decreases from the initial WCA to the stabilized WCA. Plugging Eq. 5 and Eq. 6 into Eq. 4 gives the final equation.

$$\Delta g = -(\frac{RT}{A_m}) \ln \frac{\sin\theta_{\text{sta}}}{\sin\theta_{\text{init}}} \tag{7}$$

References

(1) Extrand, C. A thermodynamic model for contact angle hysteresis. *Journal of Colloid and interface Science* **1998**, *207*, 11–19.

(2) Brochard-Wyart, F.; Quéré, D. *Capillarity and wetting phenomena: drops, bubbles, pearls, waves*; Springer, 2004.
